# Sequence-Based Characterization of Tn*5801*-Like Genomic Islands in Tetracycline-Resistant *Staphylococcus pseudintermedius* and Other Gram-positive Bacteria from Humans and Animals

**DOI:** 10.3389/fmicb.2016.00576

**Published:** 2016-04-26

**Authors:** Lisbeth E. de Vries, Henrik Hasman, Sonia Jurado Rabadán, Yvonne Agersø

**Affiliations:** ^1^Department of Technology, Metropolitan University CollegeCopenhagen, Denmark; ^2^National Food Institute, Technical University of CopenhagenLyngby, Denmark; ^3^Animal Health Department, Universidad Complutense de MadridMadrid, Spain

**Keywords:** *tet*(M), integrative and conjugative elements, ICEs, GIs, transmission, horizontal gene transfer, Tn*916*, mobile genetic elements

## Abstract

Antibiotic resistance in pathogens is often associated with mobile genetic elements, such as genomic islands (GI) including integrative and conjugative elements (ICEs). These can transfer resistance genes within and between bacteria from humans and/or animals. The aim of this study was to investigate whether Tn*5801*-like GIs carrying the tetracycline resistance gene, *tet*(M), are common in *Staphylococcus pseudintermedius* from pets, and to do an overall sequences-based characterization of Tn*5801*-like GIs detected in Gram-positive bacteria from humans and animals. A total of 27 tetracycline-resistant *S*. *pseudintermedius* isolates from Danish pets (1998–2005) were screened for *tet*(M) by PCR. Selected isolates (13) were screened for GI- or ICE-specific genes (*int*_Tn*5801*_ or *xis*_Tn*916*_) and their *tet*(M) gene was sequenced (Sanger-method). Long-range PCR mappings and whole-genome-sequencing (Illumina) were performed for selected *S*. *pseudintermedius*-isolates (seven and three isolates, respectively) as well as for human *S. aureus* isolates (seven and one isolates, respectively) and one porcine *Enterococcus faecium* isolate known to carry Tn*5801*-like GIs. All 27 *S. pseudintermedius* were positive for *tet*(M). Out of 13 selected isolates, seven contained Tn*5801*-like GIs and six contained Tn*916*-like ICEs. Two different Tn*5801*-like GI types were detected among *S. pseudintermedius* (Tn*5801* and GI*6287*) - both showed high similarity compared to GenBank sequences from human pathogens. Two distinct Tn*5801*-like GI types were detected among the porcine *E. faecium* and human *S. aureus* isolates (Tn*6014* and GI*6288*). Tn*5801*-like GIs were detected in GenBank-sequences from Gram-positive bacteria of human, animal or food origin worldwide. Known Tn*5801*-like GIs were divided into seven types. The results showed that Tn*5801*-like GIs appear to be relatively common in tetracycline-resistant *S. pseudintermedius* in Denmark. Almost identical Tn*5801*-like GIs were identified in different Gram-positive species of pet and human origin, suggesting that horizontal transfer of these elements has occurred between *S. pseudintermedius* from pets and human pathogens, including *S. aureus*.

## Introduction

The emergence of antibiotic resistance among pathogenic bacteria is a general problem which is regarded as a threat to public health in many countries – both human and veterinary infections caused by antibiotic resistant pathogens pose a challenge to treatment and have high economic costs (Marshall and Levy, 2011; Wernli et al., 2011; Wegener, 2012; U.S. Department of Health and Human Services - Centers for Disease Control and Prevention [CDC], 2013)^1^.

Resistant bacteria can develop in a human or animal host by evolutionary selection due to the presence of antibiotics, e.g., in connection with antibiotic therapy (Davies and Davies, 2010; Marshall and Levy, 2011). In addition, a host can acquire resistant bacteria by transmission from an outside source, e.g., via contact with other people and/or animals or via environmental exposure (Guardabassi et al., 2004a; Wegener, 2012; Da Costa et al., 2013). Transmission of multiple antibiotic resistant *Staphylococcus pseudintermedius* between dogs and humans has been reported (Guardabassi et al., 2004a). *S. pseudintermedius* is the most important staphylococcal pathogen in dogs where it is primarily associated with skin and ear infections, but it rarely causes infections in humans. Occasionally, this species is isolated from skin infection in cats (Bannoehr and Guardabassi, 2012).

Antibiotic resistance in pathogens is often associated with mobile genetic elements (MGEs) that can transfer antibiotic resistance genes within and between bacteria from human and/or animal hosts (Burrus et al., 2002; Bennett, 2008; Roberts and Mullany, 2011). MGEs include plasmids, transposable elements, prophages and different types of genomic islands (GIs) such as integrative and conjugative elements (ICEs) (Wozniak and Waldor, 2010).

Tn*5801*-like GIs belong to the family of Tn*916*-like ICEs. Tn*916* was originally identified in the late 1970’s as an 18-kb conjugative transposon from *Enterococcus faecalis* DS16 (Franke and Clewell, 1981). Many Tn*916*-like ICEs have a broad host range and are responsible for dissemination of the tetracycline resistance gene *tet*(M) in Gram-positive bacteria from humans and animals (Franke and Clewell, 1981; Rice, 1998; Roberts and Mullany, 2011). Tn*5801* is a putative ICE (25.3 kb), however, this has not been proven. Like Tn*916*, Tn*5801* is also associated with the *tet*(M) gene. The *tet*(M) gene encodes a ribosomal protection protein conferring resistance by protecting the ribosome against the action of tetracyclines (Chopra and Roberts, 2001). Tn*5801* was first described in a clinical *S. aureus* isolate, Mu50, from a Japanese boy (Kuroda et al., 2001). Tn*5801*-like GIs have subsequently been identified in other human *S. aureus* and *Streptococcus* isolates, mainly in pathogenic, but also in commensal strains (Kuroda et al., 2001; de Vries et al., 2009; Denapaite et al., 2010; Holden et al., 2010; Mingoia et al., 2013; Brenciani et al., 2014). Comparative genome hybridization analysis recently showed Tn*5801* to be specifically associated with human methicillin-resistant *S. aureus* (MRSA) in China (Li et al., 2013). Only a few Tn*5801*-like elements from animal-associated bacterial isolates have been reported so far: a partially sequenced element, CW459*tet*(M) in *Clostridium perfringens* isolated from porcine feces, and an element detected in an *E. faecium* isolate CICYT-205 from a healthy pig in Spain (Roberts et al., 2001; Jurado-Rabadán et al., 2014). For the latter, only *tet*(M) was sequenced. Finally, a Tn*5801*-like GI has recently been reported in a fully sequenced genome from a methicillin resistant *S. pseudintermedius* (MRSP) strain ED99 isolated from a dog in the UK (Ben Zakour et al., 2012).

The importance of GIs/ICEs associated with *tet*(M) was highlighted in a recent study. Previous extensive clinical use of tetracycline and corresponding integration of Tn*916*- and Tn*5801*-like elements in *Streptococcus agalactiae* was shown to have had great significance for the dissemination of specific pathogenic *S. agalactiae* clones in humans (Da Cunha et al., 2014).

**FIGURE 1:**
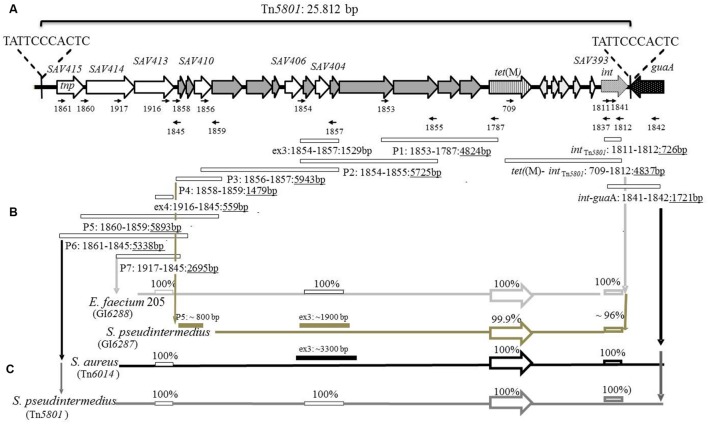
**Schematic illustration of Tn*5801* and the strategy for PCR mapping of full-length Tn*5801*-like elements. (A)** Illustration of the 25 kb putative integrative-conjugative element (ICE) integrated into the 3′end of a GMP synthetase gene *gua*A (black dotted arrow) in *Staphylococcus aureus* Mu50. Open reading frames (ORFs) are illustrated with arrows. Besides *tet*(M) (striped arrow), Tn*5801* consists of three structural domains containing genes associated with conjugation (dark gray arrows), regulation (light gray arrows), and recombination (white dotted arrow) typically of elements belonging to the Tn*916*-family. ORFs whose functions are unknown (*SAV393*, *SAV404*, *SAV406*, *SAV410*, and *SAV413*-*415*) are illustrated with white arrows. *SAV415* are predicted to encode a transposase. Direct repeats of 11 bp located in both ends of Tn*5801* are chromosomal junctions and compose a putative core site for integration of the element (Ito et al., 2003). **(B)** Schematic illustration of primers (small arrows) designed based on the Tn*5801* sequence (BA000017) and predicted PCR-products (rectangles) with expected product size listed below (see **Supplementary Tables S2** and **S3**). **(C)** Schematic illustration of results of the PCR mapping of Tn*5801*-like genomic islands (GIs) for seven pet-associated *S. pseudintermedius* isolates, seven human *S. aureus* isolates and one porcine *Enterococcus faecium* isolate.

Characteristic of MGEs, including the Tn*916*-like family, Tn*5801*-like elements have a modular organization (**Figure 1A**; Burrus et al., 2002; Toussaint and Merlin, 2002; Roberts and Mullany, 2009). Tn*5801* contains ORFs similar to those of Tn*916*, but differs by containing an integrase gene (*int*_Tn*5801*_) different from the excisionase/integrase genes (*xis*_Tn*916*_/*int*_Tn*916*_) present in *Tn916*. The *int*_Tn*5801*_- and* int*_Tn*916*_ genes are of different sizes and show very low DNA identity (<50%) – they encode different types of tyrosine recombinases. The *int*_Tn*5801*_ gene is identical to the *int459* gene from the element CW459*tet*(M) (Roberts et al., 2001). Both Tn*5801* and CW459*tet*(M) are integrated at the same site, located at the 3′end of an ORF, predicted to encode GMP synthetase, whereas Tn*916* can integrate at random sites in most hosts, but preferentially into AT-rich sequences (Burrus et al., 2002). The *tet*(M) genes from Tn*5801* and Tn*916* show high similarity (97.7% DNA identity). Only one *tet*(M)_Tn*5801*-_allele has been detected so far, and this allele appears to be specifically associated with Tn*5801*-like GIs (de Vries et al., 2009).

The overall aim of this study was to investigate whether Tn*5801*-like elements carrying *tet*(M) are common in clinical *S. pseudintermedius* isolates from pets, where they may constitute a likely reservoir for human pathogens (Guardabassi et al., 2004a,b). Another aim of the study was to perform an overall sequence-based characterization of Tn*5801*-like GIs detected in Gram-positive bacteria from humans and animals in order to get an overview of different Tn*5801*-like GI types and the degree of Tn*5801*-like GI dissemination. The study was performed by screening selected tetracycline-resistant *S. pseudintermedius* from veterinary diagnostic isolates for *tet*(M) as well as for *int* or *xis* genes specific for Tn*5801*- or Tn*916*-like elements, respectively. PCR mapping and gene/genome sequencing was performed in order to characterize and compare different Tn*5801*-like GIs detected in pet-associated *S. pseudintermedius* isolates as well as in human *S. aureus* isolates and one pig *E. faecium* isolate from previous studies (de Vries et al., 2009; Jurado-Rabadán et al., 2014). We conducted an overall comparison of full length Tn*5801*-like GIs characterized in this study and detected in genome sequences submitted to GenBank.

## Materials and Methods

### Strains

Twenty-seven tetracycline-resistant isolates from dogs and one cat were sampled and identified as *S. intermedius* by biochemical tests, morphology and 16S sequencing (**Supplementary Table S1**). Based on host origin and PCR-screening, all isolates were further identified as *S. pseudintermedius* (Bannoehr and Guardabassi, 2012). All isolates were diagnostic submissions to The National Veterinary Institute, Technical University of Denmark. Thirteen *S. pseudintermedius* isolates selected to cover the whole isolation period, 1998–2005 were characterized further in regard to *tet*(M) and Tn*5801*-like sequences (**Table 1**). In addition, one porcine *E. faecium* isolate and seven human *S. aureus* isolates identified in previous studies were also characterized (**Table 1**). All 13 *S. pseudintermedius* isolates, except one isolate, 200007910-1, were confirmed to be tetracycline-resistant by the use of Sensititre and MIC testing (Aarestrup et al., 2000a). Susceptibility MIC-testing using cefoxitin showed all 13 isolates to be methicillin sensitive *S. pseudintermedius* (MSSP). Besides tetracycline and cefoxitin, the isolates were also tested for susceptibility to penicillin, streptomycin, erythromycin, ciprofloxacin, spectinomycin, tiamulin, trimethoprim, chloramphenicol, florfenicol, gentamicin, and sulfamethoxazole by the Sensititre method as described previously (Aarestrup et al., 2000a).

**Table 1:** Selected strains with Tn*5801*-like genomic islands (GIs) characterized in this study.

Strains	Species (typing info)	AR phenotypes	Element type/[*tet*(M) type]	Source/country	Year	Reference
2001-08299-1	*S. pseudintermedius* (5-7-7-2-3-1-1)^a^	TET^R^	GI*6287*/[*te*t(M)_Tn*5801*-liketype-1_]	Dog/Denmark	2001	This study
2005-06729-1	*S. pseudintermedius*	PEN^R^ SPE^R^ TET^R^	GI*6287*/[*te*t(M)_Tn*5801*-liketype-1_]	Dog/Denmark	2005	This study
98-41998-1	*S. pseudintermedius* (1-3-2-20-11-1-1)^a^	ERY^R^ PEN^R^ SPE^R^ SMX^R^ STR^R^TET^R^	Tn*5801*/[*te*t(M)_Tn*5801*_]	Cat/Denmark	1998	This study
99-07249-2	*S. pseudintermedius*	CHL^R^ERY^R^ PEN^R^STR^R^TET^R^	Tn*5801*/[*te*t(M)_Tn*5801*_]	Dog/Denmark	1999	This study
98-41787-1	*S. pseudintermedius* (1-3-2-20-11-1-1)^a^	CHL^R^ERY^R^ PEN^R^STR^R^TET^R^	Tn*5801*/[*te*t(M)_Tn*5801*_]	Dog/Denmark	1998	This study
2005-06768-1	*S. pseudintermedius*	CHL^R^ERY^R^ PEN^R^STR^R^TET^R^	Tn*5801*/[*te*t(M)_Tn*5801*_]	Dog/Denmark	1998	This study
2003-07869-1	*S. pseudintermedius*	PEN^R^TET^R^	Tn*5801*/[*te*t(M)_Tn*5801*_]	Dog/Denmark	2003	This study
2000-7910-1	*S. pseudintermedius*^b^	CHL^R^ERY^R^ PEN^R^STR^R^TMP^R^	T*n916*-like ICE/[*tet*(M)_Tn_*_*916*_*_-like_ _type-1_]	Dog/Denmark	2000	This study
2001-08127-3	*S. pseudintermedius*	SMX^R^TET^R^	T*n916*-like ICE/[*tet*(M)_Tn_*_*916*_*_-liketype-2_]	Dog/Denmark	2001	This study
2003-07768-1	*S. pseudintermedius*	SMX^R^TET^R^	T*n916*-like ICE/[*tet*(M)_Tn_*_*916*_*]	Dog/Denmark	2003	This study
2001-08050-1	*S. pseudintermedius*	ERY^R^PEN^R^ STR^R^TET^R^	T*n916*-like ICE/[*tet*(M)_Tn_*_*916*_*]	Dog/Denmark	2001	This study
2005-06416-1	*S. pseudintermedius*	CHL^R^ERY^R^ PEN^R^STR^R^TET^R^ TMP^R^	T*n916*-like ICE/[*tet*(M)_Tn_*_*916*_*]	Dog/Denmark	2005	This study
99-06237-1	*S. pseudintermedius*	ERY^R^ PEN^R^STR^R^TET^R^ TMP^R^	T*n916*-like ICE/[*tet*(M)_Tn_*_*916*_*]	Dog/Denmark	1999	This study
CICYT-205	*E. faecium* (1-9-5-1-1-20-3/ST 437)^a^	ERY^R^ KAN^R^MXF^R^ STR^R^ TET^R^	GI*6288*/[*te*t(M)_Tn*5801*_]	Pig/Spain	2005	Jurado-Rabadán et al., 2014
213	*S. aureus* (t008/CC8)	PEN^R^STR^R^	Tn*6014*/[*te*t(M)_Tn*5801*_]	Human/Denmark	1957	de Vries et al., 2009
229	*S. aureus* (t008/CC8)	PEN^R^STR^R^	Tn*6014*/[*te*t(M)_Tn*5801*_]	Human/Denmark	1957	de Vries et al., 2009
1680	*S. aureus* (t051/CC8)	ERY^R^MET^R^PEN^R^STR^R^	Tn*6014*/[*te*t(M)_Tn*5801*_]	Human/Denmark	1963	de Vries et al., 2009
1742	*S. aureus* (t008/CC8)	PEN^R^STR^R^	Tn*6014*/[*te*t(M)_Tn*5801*_]	Human/Denmark	1963	de Vries et al., 2009
33597	*S. aureus* (t037/CC8)	CIP^R^ERY^R^GEN^R^ MET^R^PEN^R^STR^R^	Tn*6014*/[*te*t(M)_Tn*5801*_]	Human/Denmark	2000	de Vries et al., 2009
34148	*S. aureus* (t037/CC8)	CIP^R^ERY^R^GEN^R^ MET^R^PEN^R^STR^R^	Tn*6014*/[*te*t(M)_Tn*5801*_]	Human/Denmark	2000	de Vries et al., 2009
34168	*S. aureus* (t037/CC8)	CIP^R^ERY^R^GEN^R^ MET^R^PEN^R^STR^R^	Tn*6014*/[*te*t(M)_Tn*5801*_]	Human/Denmark	2000	de Vries et al., 2009

*CHL, chloramphenicol; CIP, ciprofloxacin; ERY, erythromycin; GEN, gentamicin, KAN, kanamycin; MET, methicillin, MXF, moxifloxacin; PEN, penicillin; SMX, sulfamethoxazole; SPT, Spectinomycin; STR, streptomycin; TMP, trimethoprim; ^*R*^, resistant; ST, sequence type; MLST, multi locus sequence type; AR, antibiotic resistance.^a^ MLST allelic profile and ST type (if known) determined by submitting assembled contigs to MLST typing tool (1.7) from Center for Genomic Epidemiology*^2^*(Larsen et al., 2012).^b^MIC-testing showed this isolate to have an MIC for tetracycline ≤0,5 μl/ml and was therefore not confirmed to be TET^*R*^. The *tet*(M)_Tn_*_*916*_*_*-like type 1*_ gene contained 3 bp deletion compared to *tet*(M)_*Tn*_*_*916*_* and *tet*(M)_*Tn*_*_*916*_*_*-like type 2*_ which may explain the lack of TET^*R*^ phenotype.*

### PCR Screening and Sequencing of *tet*(M), *int*_Tn*5801*_ and *xis*_Tn*916*_ in *S. pseudintermedius*

All 27 *S. pseudintermedius* isolates were screened for *tet*(M) by PCR as described previously (see **Supplementary Table S2**; Aarestrup et al., 2000b). Thirteen *tet*(M)-positive isolates (**Table 1**) were selected for *tet*(M)-gene-sequencing by the Sanger method in a manner similar to a previously described strategy (see Supplementary Information; de Vries et al., 2009). In addition, the 13 isolates were screened for *int*_Tn*5801*_ or *xis*_Tn_*_*916*_* genes by PCR, and selected screening products were sequenced by the Sanger method and compared to sequences from Tn*5801* and Tn*916* as described in former studies (Agerso et al., 2002; de Vries et al., 2009). (See Supplementary Information, including **Supplementary Tables S2** and **S3** for further details).

### PCR Mapping of Tn*5801*-Like GIs from Pet-Associated *S. pseudintermedius*, Human *S. aureus* and a Porcine *E. faecium*

The strategy for mapping the Tn*5801*-like elements is outlined in **Figures 1A,B**. Elements detected in pet-associated *S. pseudintermedius* from this study and previously detected elements in isolates from human *S. aureus* and porcine *E. faecium* were mapped (de Vries et al., 2009; Jurado-Rabadán et al., 2014). The mapping was performed by Long range PCR with the Phusion^TM^ High-Fidelity DNA Polymerase (Finnzymes, Finland) using the conditions recommended by the manufacturer. DNA Taq polymerase (Ampliqon, Denmark) was used to obtain PCR products expected to be <2000 bp. The PCR conditions and primers are listed in **Supplementary Tables S2** and **S3**. *S. aureus* Mu50 was used as positive control and a *S. pseudintermedius* strain 200307768-1 that was shown to be negative for *int*_Tn*5801*_ and positive for *xis*_Tn_*_*916*_*, was used as negative control. Besides *int*_Tn*5801*_, selected PCR-products, ex3 and ex4, were fully or partially sequenced by the Sanger method and compared to corresponding sequences in Tn*5801*.

### Whole-Genome Sequencing of Selected *S. pseudintermedius*, *S. aureus*, and *E. faecium*

Based on the PCR mapping of detected Tn*5801*-like elements and *tet*(M) sequence types 3 *S. pseudintermedius* strains (2001-08299-1, 9841998-1, and 9841787-1), *S. aureus* 1680 and *E. faecium* CICYT-205 were selected for whole-genome sequencing. Genomic DNA was purified by using the Easy-DNA gDNA Purification kit (Life Sciences). For each isolate, a sequencing library was constructed and pooled using Nextera DNA Sample Preparation Kit (Illumina). Sequencing was performed using the Illumina platform, MiSeq (paired-end reads). Paired-end reads were trimmed directly by the MiSeq in order to remove sequencing adaptor and primer contamination, and CLC Genomic Workbench (version 7.5.1) was used to remove low quality reads. *De novo* assembly was conducted using CLC Genomic Workbench (version 7.5.1). (See Supplementary Information including **Supplementary Tables S4** and **S5** for details regarding trimming and assembly). For all five isolates, assembled contigs of minimum 500 bp (for CICYT-205 cut-off was minimum 1000 bp contigs) with an average coverage >30x were submitted as a Whole Genome Shutgun (WGS) sequencing project to GenBank under bioProject: PRJNA264198; bioSamples: SAMN03120277, SAMN03120291, SAMN03120292, SAMN03120293, SAMN03120290; accession no: JTKN00000000, JTKO00000000, JTKP00000000, JTKQ00000000, JTKR00000000. The WGS submissions were annotated by NCBI Prokaryotic Genome Automatic Annotation Pipeline (PGAAP)^3^ (Angiuoli et al., 2008).

### Bioinformatic Analysis of Sequenced Genomes with Focus on Tn*5801*-Like GIs

For all the five WGS-sequences, species were confirmed by using the strain identification tool (EzTaxon server)^4^ and/or the species finder tool (SpeciesFinder 1.0)^5^ using annotated full length 16S rRNA sequences and/or assembled contigs as input, respectively (Larsen et al., 2014). Besides, all five WGS-sequences were analyzed for MLST allelic profile, ST type and the presence of antibiotic resistance genes using the online-tools MLST typing tool (1.7) and ResFinder^6^ (**Table 1** and **Supplementary Table S6**) (Zankari et al., 2012). Contigs containing Tn*5801*-like *tet*(M) and *int*_Tn*5801*_ sequences from the sequenced genomes were detected by search annotations for “*tet*(M)” and “*int”* as well as sequences for identity to *int*_Tn*5801*_. For all staphylococci isolates a Tn*5801*-like element was detected in one contig, whereas for the *E. faecium* CICYT-205 isolate, specific Tn*5801*-like sequences were detected in two contigs that showed an overlap of 129 bp with 100% DNA identities. The two contigs were fused in Geneious (Trial version 8.0.4). Primers used in the PCR mapping were mapped to the selected contigs containing Tn*5801*-like elements, and expected PCR-product sizes were compared to product sizes obtained in the PCR mapping in order to verify correct assembly. Sequence analysis was performed using CLC Genomic Workbench (version 7.5.1 or 8.5.1). GenBank was searched for full length Tn*5801*-like GIs based on a BLASTp^7^ search using the annotated Int protein of Tn*5801* as query (GenBank accession no. BAB56554). A threshold of minimum 98% amino acid (aa.) identity was used, and 11 full-length elements for which sequences were published were selected for comparative sequence analysis together with elements sequenced in this study.

## Results

### The *tet*(M) Gene in *S. pseudintermedius* from Pets Was Detected on Tn*5801*- and Tn*916*-Like Elements

Twenty seven tetracycline-resistant clinical *S. pseudintermedius* isolates were screened for the presence of the *tet*(M) gene. All 27 isolates were positive for the *tet*(M)-PCR screening (**Supplementary Table S1**). Of the 13 sequenced *tet*(M) genes, seven were highly similar to *tet*(M)_Tn*5801*_ from *S. aureus* Mu50 (99.9–100% DNA identity), and six sequences were highly similar to *tet*(M)_Tn_*_*916*_* from *E. faecalis* DS16 (99.8–100% DNA identity). Altogether two Tn*5801*-like *tet*(M)-sequence types were detected: *te*t(M)_Tn*5801*_ and *tet*(M)_Tn*5801*-like-type-1_ differing by 1/1920 bp. In addition, 3 Tn*916*-like-*tet*(M) sequence types were detected: *tet*(M)_Tn_*_*916*_*, *tet*(M)_Tn_*_*916*-like-_*_type-1_ and *tet*(M)_Tn_*_*916*-like-_*_type-2_ differing by 1–3/1920 bp. (See **Table 1** and **Supplementary Table S7** for details). PCR screenings confirmed that the 13 tested strains contained *int*_Tn*5801*_ or *xis*_Tn_*_*916*_* genes. In addition, long PCR confirmed that the 7 Tn*5801*-like *tet*(M) genes were physically linked to detected *int*_Tn*5801*_ genes (see **Figures 1B,C**).

### Two Different Tn*5801*-Like GIs Types Were Detected Among the *S. pseudintermedius*

**FIGURE 2:**
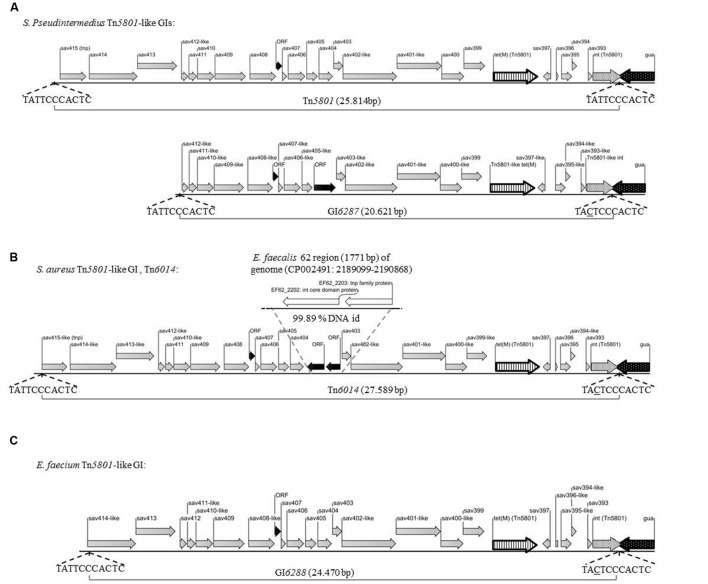
**Illustration of Tn*5801*-like GIs from Gram-positive bacteria fully sequenced in this study**. Gray arrows illustrate ORFs identical (*sav393*-*sav397*, and *sav399-sav415*) or similar (*sav393*-like-*sav415*-like) to corresponding ORFs in Tn*5801*, black arrows illustrate ORFs which were not annotated in Tn*5801*. Striped arrow: *tet*(M), white dotted arrow: Tn*5801*-like integrase gene (*int*), black dotted arrow: *gua*A, *tnp*: tranposase. Direct repeats of 11 bp located in both ends of Tn*5801*-like elements are chromosomal junctions and compose a putative core site for integration of the element (Ito et al., 2003). Underlined nucleotides differ from Tn*5801*. **(A)** Tn*5801* from *S. pseudintermedius* 9841787-1 isolated from a dog and 9841998-1 isolated from a cat – sequenced GIs from the two isolates were 100% identical. GI*6287* from *S. pseudintermedius* 200108299-1 isolated from a dog. **(B)** Tn*6014* from human *S. aureus* 1680 **(C)** GI*6288* sequenced from *E. faecium* CICYT205.

PCR mapping and whole genome sequencing revealed two different Tn*5801*-like elements among the tested *S. pseudintermedius* isolates corresponding to the two different Tn*5801*-like *tet*(M) types detected (see **Figures 1** and **2A**).

For 5 *S. pseudintermedius* isolates, the PCR mapping showed that all amplified PCR products were of the same size compared to the control strain Mu50 (**Figures 1B,C** and **Table 1**). Besides *tet*(M)_Tn*5801*_, the *int*_Tn*5801*_ screening product and sequenced ex4- and ex3 fragments from these strains were shown to be 100% identical to corresponding regions in Tn*5801* (**Figures 1B,C**). The whole element present in theses isolates was shown to be almost identical to Tn*5801* from *S. aureus* Mu50 both in organization as well as on DNA sequence level (**Table 2**). In addition, all ORFs of this element were identical or highly similar to Tn*5801* ORFs (*int*_Tn*5801*_-*sav*415; **Figure 2A**).

**Table 2:** Comparison of whole Tn*5801*-like GI-sequences from this study with corresponding sequences from Tn*5801*, *S. aureus* Mu50.

GI	Tn*5801*		
	Identical bp^a^/total bp^b^	DNA identity^a^ (%)	Gaps (number of gaps^a^/total bp^b^)
*S. pseudintermedius* Tn*5801*	25807/25814	99.97	2/25812
*S. pseudintermedius* GI*6287*	19172/25812	73.12	6009/25812
*S. aureus* Tn*6014*	25782/27589	93.45	1777/27589
*E. faecium* GI*6288*	24451/25812	94.72	1346/25812

^a^*Determined by pairwise alignment and comparison using CLC Genomic Workbench (version 8.5.1)*.

^b^*Corresponding to the largest of the compared GIs*.

For two other *S. pseudintermedius* strains, the PCR mappings were positive, except for *int*-*gua*A, ex4, P6 and P7. The size of P2, P3, ex3, and P5 differed from Tn*5801* (**Figures 1B,C**). These strains contained the *tet*(M)_Tn*5801*-like-type_*_-1_* gene. Compared to Tn*5801*, the *int*_Tn*5801*-like_ screening product showed 96% DNA identity. This element showed similar organization compared to Tn*5801*, but differed by being approximately 5000 bp smaller than Tn*5801* corresponding to a missing upstream region containing *sav413*-*sav415* of Tn*5801* (**Figure 2A** and **Table 2**). Instead of Tn*5801 sav404*, this element contained a larger ORF with low similarity to *sav404*. Thus, this element was registered as a novel element, GI*6287* in the Transposon Nomenclature Database from the UCL Eastman Dental Institute, London^8^ (Roberts et al., 2008; Roberts and Mullany, 2011).

Both *S. pseudintermedius* Tn*5801* and GI*6287* were found to be located in the 3′end of putative GMP synthase genes (*guaA*) with high similarity (99.68% DNA identity) and downstream of a putative integrase pseudogene with high similarity (97.45% DNA identity). Both elements are flanked by 11 bp sequences that are almost identical to the direct repeats that form the core of the putative attachment sites of Tn*5801* (Ito et al., 2003; **Figure 2A**).

### Two Other Tn*5801*-Like GI Types Were Characterized from the Human *S. aureus* Isolates and One *E. faecium* Isolate from Pig

Characterization of Tn*5801*-like GIs previously detected in human clinical *S. aureus* from Denmark and an *E. faecium* strain isolated from a healthy pig in Spain revealed 2 other Tn*5801*-like GI types different from the elements detected among the *S. pseudintermedius* isolates (see **Figures 1** and **2**; de Vries et al., 2009; Jurado-Rabadán et al., 2014).

The PCR mapping showed that for the seven *S. aureus* isolates, PCR fragment P1, P4, ex4, P5-P7 were of the same size as Tn*5801* (**Figures 1B,C**). PCR products P2, P3, and ex3 were approximately 1.7-kb longer than the corresponding fragments detected in Mu50. As for *tet*(M)_Tn*5801*_, the *int*_Tn*5801*_ screening product and the ex4 fragment showed 100% DNA identity with Tn*5801*. This element is referred to as Tn*6014* as previously registered (de Vries et al., 2009). The whole Tn*6014* sequence contained identical or highly similar ORFs and showed overall similarity compared to Tn*5801* (*int*_Tn*5801*_-*sav*415; **Figure 2B** and **Table 2**). In addition, Tn*6014* contained an additional region of 1775 bp corresponding to two putative ORFs not present in Tn*5801*.

For the *E. faecium* isolate, all PCRs except for P6, P5 and *int*-*gua*A were shown to be of the same size as for Tn*5801* (**Figures 1B,C**). Besides the *tet*(M)_Tn*5801*_ gene sequenced previously (Jurado-Rabadán et al., 2014), the *int*_Tn*5801*_ screening product and the ex3 fragment showed 100% DNA identity with Tn*5801*. This element showed similarity on DNA level and a similar organization compared to Tn*5801*, except that it did not contain a *sav*415-like ORF that is predicted to encode a transposase in Tn*5801* (**Figure 2C** and **Table 2**). This element was registered as a novel element: GI*6288*.

Both Tn*6014* and GI*6288* were found integrated into the 3′ end of *guaA*. The 11-bp sequences that correspond to the imperfect direct repeats included in the *attL* and *attR* sites that flank the elements were almost identical to those of Tn*5801* (see **Figures 2B,C**).

### Sequence Analysis of *S. aureus* Tn*6014*

Further sequence analysis (BLASTn search in GenBank)^9^ of Tn*6014* from the human *S. aureus* strain showed that most of the additional 1775 bp-region detected in Tn*6014* was highly similar (99.89% DNA identity) to a region containing ORFs predicted to encode an integrase and a transposase in the genome of an *E. faecalis* strain 62 isolated from a stool sample of a healthy Norwegian infant (Brede et al., 2011). This isolate was reported to contain a Tn*916* element at a different location (accession no. CP002491; 562138–582758) than the 1771 bp region similar to a region in Tn*6014*. However, we showed it to be a Tn*5801*-like element containing *int*_Tn*5801*_ integrated into a *gua*A gene (see next paragraph). Thus, Tn*6014* is shown to be a composite element that may have been formed within Gram-positives species, possibly *Enterococcus*.

### Comparison of Tn*5801*-Like GIs Shows Evidence of Horizontal Transfer between Gram-positive Species of Human and Animal Origin

**FIGURE 3:**
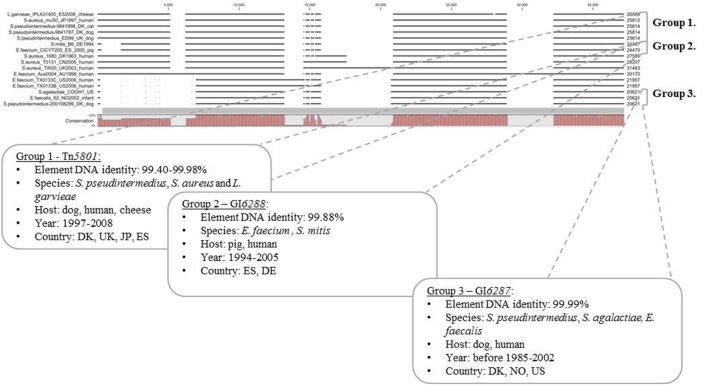
**Comparison of full length Tn*5801*-like GIs from this study (5) and full-length Tn*5801*-like elements detected in sequences from GenBank (11)**. A multiple alignment of selected Tn*5801*-like sequences constructed and visualized with CLC (version 7.5.1) revealed overall seven Tn*5801* GI types based on similar organization and DNA identity >99%. Element sizes are shown at the end of the sequences (to the right). EFA, *E. faecalis;* SAG, *Streptococcus agalactiae*; SA, *Staphylococcus aureus*; SP: LG, *Lactococcus garvieae*; SM, *Streptococcus mitis*. (EF_TX0133C/Bahaman: two strains TX0133C and TX0133B).

Tn*5801*-like GIs were found to be present in GenBank sequences from different Gram-positive bacterial pathogens and a few commensals, mainly from humans, but also sequences from bacteria associated with animals and food in Europe, USA, Asia, and Australia were found (see **Supplementary Table S8**). A multiple alignment of 11 selected full-length Tn*5801*-like GIs from GenBank and the five Tn*5801*-like GIs characterized in this study divided the elements into seven predicted GI types. All Tn*5801*-like GIs characterized in this study except for Tn*6014* fell into three different groups (groups 1–3; **Figure 3**). Pairwise comparisons of full-length elements within groups 1, 2, and 3 showed very high similarity. In addition to *S. aureus* Tn*5801*, *S. pseudintermedius* Tn*5801* showed very high similarity to the Tn*5801*-like element from canine *S. pseudintermedius* ED99 within group 1 (99.98%). Besides, *S. pseudintermedius* Tn*5801* showed high similarity to an element from a *Lactococcus garvieae* IPLA31405 strain isolated from cheese (99.40% DNA identities). Within group 2, *E. faecium* GI*6288* showed 99.88% DNA identity compared to an element from a human *S. mitis* B6 isolate. Within group 3, *S. pseudintermedius* GI*6287* was shown only to differ by 4 out of 20,621 bp from corresponding elements in human isolates, *E. faecalis* strain 62 and *S. agalactiae* COOH1. This comparison shows that almost identical full-length Tn*5801*-like GIs are present in Gram-positive species of animal and human origin, which strongly supports the occurrence of recent direct or indirect horizontal transfer of these elements between different species of different origin. These results in particular support occurrence of horizontal transfer of Tn*5801* and GI*6287* between pet-associated *S. pseudintermedius* and Gram-positive pathogens of human origin.

## Discussion

Tetracycline resistance is common in *S. pseudintermedius* and is most often mediated by the *tet*(M) gene (Schwarz et al., 1998; Kim et al., 2005). In agreement with this, we found all the tetracycline-resistant *S. pseudintermedius* isolates in this study (27) to contain the *tet*(M) gene. We cannot completely rule out that tetracycline resistance could also partly be mediated by other genes, as this was not further investigated. However, this is unlikely, since no other tetracycline resistance genes were detected by the sequence analysis of the fully sequenced *S. pseudintermedius* genomes (**Supplementary Table S6**). For the thirteen isolates for which the *tet*(M) gene was sequenced, we showed that six and seven contained Tn*916*-like and Tn*5801*-like elements, respectively. This shows Tn*5801*-like GIs to be relatively common in tetracycline-resistant pet-associated *S. pseudintermedius* in Denmark, and the prevalence appears to be on the same level as for Tn*916*-like ICEs. Previously, Tn*5801*-like GIs (Tn*6014*) were detected in 7 out of 20 *tet*(M)-positive human *S. aureus* isolates from Denmark (1957–2001; de Vries et al., 2009). As opposed to this, 17 porcine *S. aureus* strains from the same study only contained *tet*(M) associated with Tn*916*–like ICEs. A Tn*5801*-like element was recently reported in a MRSP canine strain (Schwarz et al., 1998; Ben Zakour et al., 2012). However, this study is the first to address the prevalence of Tn*5801*-like elements among tetracycline-resistant *S. pseudintermedius* and the first report on Tn*5801*-like elements within MSSP.

Previous studies have suggested that specific *tet*(M)-alleles or subtypes of *tet*(M) are associated with a specific element type or element subgroups within the Tn*916*-like ICE family as well as other ICE families (Agerso et al., 2006; de Vries et al., 2009; Jurado-Rabadán et al., 2014). Among the tested *S. pseudintermedius* isolates with Tn*5801*-like GIs two different subtypes were detected corresponding with two highly similar *tet*(M) sequence types that only differed by 1/1920 bp corresponding to a difference of 1/640 aa. The two *tet*(M) subtypes were shown both by Sanger and Illumina sequencing. This is the first report of a new *Tn5801*-like *tet*(M) subtype. This subtype may be specifically associated with GI*6287*, however, the almost identical Tn*580*1-like GIs that are present in *E. faecalis* strain 62 and *S. agalactiae* COOH1 contain the known Tn*5801*-*tet*(M) – provided that the difference does not result from a sequencing mistake.

Tn*5801* was detected in the genome of *S. pseudintermedius* isolates from four dogs and a one cat, including two isolates of the same unknown sequence type (ST). *S. pseudintermedius* Tn*5801* was shown to be almost identical compared to the element detected in the MRSP *S. pseudintermedius* ED99 (UK, ST type 25) and the Tn*5801* element from the Japanese human *S. aureus* strain (Mu50). As mentioned above, *S. pseudintermedius* GI*6287* was shown to be highly similar to elements present in human *Streptococcus* and *Enterococcus* strains from US and Norway, respectively. The fact that almost identical GIs can be found in different species of animal and human pathogens supports that horizontal transfer of these elements has occurred between the *S. pseudintermedius* and human pathogens – the direction of transfer cannot be determined, however. Alternatively, the different species may have received these elements from a common bacterial source.

The mechanism of transfer for Tn*5801* is unknown, but the similar organization of Tn*5801* and Tn*916* may suggest that Tn*5801*, like Tn*916*, transfer horizontally by conjugation with a rolling circle (Rice, 1998; Mullany et al., 2002). The only evidence of horizontal transfer of an Tn*5801*-like element is presented in a previous study (de Vries et al., 2009). Tn*6014* from human *S. aureus* strain 1680 was shown to transfer at very low frequencies to a recipient strain - actually only one transconjugant was shown to have received the Tn*6014*-*tet*(M) gene physically linked to the *int*_Tn*5801*_. Attempts to detect proof of circularization of Tn*6014* by PCR did not succeed (data not shown). In this study we were not able to transfer Tn*5801*-like elements from *S. pseudintermedius* 200108299-1 or 9841998-1 to recipient strains, *E. faecalis* JH2-2, *E. faecium* BH4105, *S. aureus* 8794RF or *S. aureus* 4220RF, by filter-mating (data not shown). This was also the case in a study reporting a Tn*5801*-like element from *S. agalactiae* (Tn*5801.Sag*), for which circularization and conjugal transfer could not be shown (Mingoia et al., 2013). Apparently, horizontal transfer of Tn*5801*-like GIs occurs at extremely low frequencies and/or under highly specific conditions. These elements do not contain *xis*-like genes which are required for excision. Thus Tn*5801*-like elements are most likely mobilizable genomic islands (MGIs) that need a helper element providing them with a factor allowing their excision (Bellanger et al., 2014). Tn*5801*-like elements could also turn out to be true ICEs that encode their own excision, transfer by conjugation and their integration. However, we can not rule out that these elements may have spread in bacterial populations by transformation or transduction.

Overall, different types of Tn*5801*-like GIs were found to be present in publicly available sequences from *Enterococcus*-, *Lactobacillus*-, *Lactococcus*-, *Staphylococcus*-, *Streptococcus*-, and *Clostridium* species associated with humans, animals or foods in Europe, USA, Asia, and Australia. This does show that successful dissemination of different types of this element has occurred among Gram-positive species world-wide. The observed diversity among the Tn*5801*-like GIs suggests plasticity of this element-type within Gram-positives, which is supported by the characterization of Tn*6014* in *S. aureus* - Tn*6014* contains a smaller region with a predicted integrase and a transposase gene, which may derive from *Enterococcus*.

Tn*5801*-like GIs can be dated back to the 1950’s in human *S. aureus* (de Vries et al., 2009). In *Enterococcus*, a Tn*5801*-like GI is present in the human *E. faecium* E1636 strain isolated in 1961 (**Supplementary Table S8**; van Schaik et al., 2010). This is not many years after tetracycline resistance was first observed, and considering the complexity of these GIs it is highly unlikely that these elements should have evolved within only a few years. Thus, Tn*5801*-like (and Tn*916*-like) elements may have evolved long before tetracycline was introduced into the clinical practice and have transferred into human pathogenic species early in the so called “antibiotic era.” Tn*5801*-like GIs could also have existed in human pathogens or reservoirs of pathogens prior to the “antibiotic era” and evolved by acquiring the *tet*(M) gene.

## Conclusion

This study presents evidence which supports that pet-associated *S. pseudintermedius* is a likely reservoir of Tn*5801*-like GIs detected in human pathogens (e.g., *S. aureus*). However, larger full-genome sequencing and metagenomic sequencing studies of animal-associated isolates/samples from an expanded time-period (including the pre-antibiotic era) should be conducted in order to make a final conclusion regarding the origin of Tn*5801*-like GIs in human pathogens such as *S. aureus*. Besides, the mechanism of transfer for Tn*5801*-like elements remains to be shown.

.

## Author Contributions

LV and YA designed the study and did most analysis as well as data interpretation of the work. HH contributed substantially to analysis and data interpretation regarding the genome sequencing. SR did experimental work and data interpretation regarding PCR-screenings. LV drafted the paper and all co-authors (YA, HA, and SR) have critically revised it.

## Conflict of Interest Statement

The authors declare that the research was conducted in the absence of any commercial or financial relationships that could be construed as a potential conflict of interest.

## Footnotes

1 Centers for Disease Control and prevention (CDC): http://.cdc.gov/features/antibioticresistance/

2 Center for Genomic Epidemiology MLST-typing tool: MLST 1.7 (MultiLocus Sequence Typing): https://cge.cbs.dtu.dk//services/MLST/

3 PGAAP: http://www.ncbi.nlm.nih.gov/genome/annotation_prok

4 E. Strain identification tool: http://www.ezbiocloud.net/eztaxon

5 Center for Genomic Epidemiology SpeciesFinder 1.0 Server: https://cge.cbs.dtu.dk/services/SpeciesFinder/

6 Center for Genomic Epidemiology Antibiotic resistance finder tool: https://cge.cbs.dtu.dk//services/ResFinder/

7 NCBI BLAST. http://blast.ncbi.nlm.nih.gov/Blast.cgi

8 http://www.ucl.ac.uk/eastman/tn/

9 NCBI BLAST. http://blast.ncbi.nlm.nih.gov/Blast.cgi
