# Supplementary material for: Sequence-Based Characterization of Tn5801-Like Genomic Islands in Tetracycline-Resistant Staphylococcus pseudintermedius and Other Gram-positive Bacteria from Humans and Animals
Source: Front Microbiol. 2016 Apr 26;7:576. doi: 10.3389/fmicb.2016.00576 (PMC4844618; doi:10.3389/fmicb.2016.00576)
Supplement: Supplementary file 1 [file Table_1.DOCX]

***Supplementary information***

## Sequence-based characterization of Tn5801-like genomic islands in tetracycline-resistant Staphylococcus pseudintermedius and other Gram-positive bacteria from humans and animals

*Lisbeth Elvira de Vries*, Henrik Hasman, Sonia Jurado Rabadán and Yvonne Agersø*

* Correspondence:

Metropolitan University College

Department of Technology

Sigurdsgade 26

2200 Copenhagen, Denmark

[lidv@phmetropol.dk](mailto:lidv@phmetropol.dk)

**Index**

[Sequencing of tet(M) from thirteen S. pseudintermedius isolates 1](#_Toc441483610)

[Genome sequencing details – trimming and assembly 2](#_Toc441483611)

[Supplementary Tables 3](#_Toc441483612)

[Supplementary Table S1: 3](#_Toc441483613)

[Supplementary Table S2: 4](#_Toc441483614)

[Supplementary Table S3: 5](#_Toc441483615)

[Supplementary Table S4: 5](#_Toc441483616)

[Supplementary Table S5: 6](#_Toc441483617)

[Supplementary Table S6: 6](#_Toc441483618)

[Supplementary Table S7: 6](#_Toc441483619)

[Supplementary Table S8: 7](#_Toc441483620)

[References 8](#_Toc441483621)

###

### Sequencing of tet(M) from thirteen S. pseudintermedius isolates

Two or four overlapping PCR fragments covering *tet*(M) were amplified and used as template for sanger sequencing.(de Vries et al., 2009) All sanger sequencing was performed by Macrogen, Korea.(Macrogen, 2008) For six isolates (99-06237-1, 2000-07910-1, 2001-08050-1, 2001-08127-3, 2003-07768-1, 2005-06416-1) primer pairs 526-323 and 307-1756 were used to amplify two overlapping PCR fragments covering *tet*(M) and primers 526, 540, 709, 525, 307, 323 and 1756 were used for sequencing (primers are described previously(de Vries et al., 2009) and some are listed in Supplementary Table S3). For seven isolates (98-41787-1, 98-41998-1, 99-07249-2, 2001-08299-1, 2003-07869-1, 2005-06729-1, 2005-06768-1) primer pairs 526-540, 324-525, 266-323 and 307-1756 were used to amplify four overlapping PCR fragments and all amplification primers except for 526 were used for sequencing (Supplementary Table S3). DNA Taq polymerase (Amplicon, Denmark) was used for all PCR amplifications.

## Genome sequencing details – trimming and assembly

CLC Genomic Workbench (version 7.5.0 or 7.5.1) was used for trimming of paired-end reads and de novo assembly. Default parameters were used for the trimming (Quality trimming – trim using quality score limit 0,05; trim ambiguous nucleotides – Maximum number of ambiguities: 2. Trim bases: 1; Filter on length: discard reads below length 15, above 1000). For the de novo assembly the following parameters were used: automatic word size, word size: 20; automatic bubble size, bubble size: 50, minimum contig length 500 bp (all the staphylococci sequences) or 1000 bp (the enterococcus sequences); auto-detect paired distance; perform scaffolding; Map reads back to contigs (slow), Mismatch cost: 2, Insertion cost: 3, Deletion cost: 3, Length fraction: 0,5; Similarity fraction: 0,8. The *E. faecium* CICYT-205 isolate was known to contain two different *tet*(M) alleles.(Jurado-Rabadán et al., 2014) Thus, in order to get a correct assembly of the two *tet*(M) genes, selected PCR fragments from the PCR-mapping were fully or partial sequences by the Sanger Method (Macrogen, Korea) using PCR-primers as sequencing primers. These sequences (see Supplementary Table S4 below) were used in the assembly with “Guidance only reads” option. Results for the assembly are listed in Supplementary Table S5.

## Supplementary Tables

Supplementary Table S1: Tetracycline resistant *S. pseudintermedius* isolates from Denmark screened for *tet*(M)

| *S. pseudintermedius* strain | Year | Host | Source |
| --- | --- | --- | --- |
| 98-41787-1^*§^ | 1998 | dog | skin |
| 98-41996-1 | 1998 | dog | urine |
| 98-41998-1^*§^ | 1998 | cat | skin |
| 99-06237-1^*^ | 1999 | dog | nail |
| 99-06369-1 | 1999 | dog | ear |
| 99-06539-1 | 1999 | dog | trachea |
| 99-07063-1 | 1999 | dog | eye |
| 99-07249-2^*^ | 1999 | dog | skin |
| 2000-07151-1 | 2000 | dog | vagina |
| 2000-07910-1^*^ | 2000 | dog | urine |
| 2001-07968-1 | 2001 | dog | ear |
| 2001-08050-1^*^ | 2001 | dog | skin |
| 2001-08127-3^*^ | 2001 | dog | vagina |
| 2001-08155-1 | 2001 | dog | skin |
| 2001-08299-1^*§^ | 2001 | dog | nail |
| 2002-06216-1 | 2002 | dog | nose |
| 2002-06690-1 | 2002 | dog | skin |
| 2002-06949-1 | 2002 | dog | pus |
| 2003-07065-1 | 2003 | dog | ear |
| 2003-07768-1^*^ | 2003 | dog | swab |
| 2003-07869-1^*^ | 2003 | dog | ear |
| 2004-06179-1 | 2004 | dog | furuncle |
| 2004-06372-1 | 2004 | dog | skin |
| 2004-06493-1 | 2004 | dog | skin |
| 2005-06416-1^*^ | 2005 | dog | furuncle |
| 2005-06729-1^*^ | 2005 | dog | boil |
| 2005-06768-1^*^ | 2005 | dog | pus |

* isolates selected for sequencing of *tet*(M)

§ isolates selected for whole genome sequencing

Supplementary Table S2: PCR conditions for PCR-screenings and mapping of Tn*5801*-like elements from *S. pseudintermedius*, *S. aureus* and *E. faecalis*

| PCR product | Primers | PCR conditions |
| --- | --- | --- |
| *int*-*gua*A | 1841,1842 | 30 s at 98**°**C followed by 30 cycles of 10 s at 98**°**C, 30 s at 61**°**C and 52 s at 72**°**C, and a final extension for 10 min at 72**°**C(3 min at 94**°**C followed by 30 cycles of 30 s at 94**°**C, 30 s at 54**°**C and 120 s at 72**°**C, and a final extension for 10 min at 72**°**C)* |
| *int*1 | 1811,1812 | (3 min at 94**°**C followed by 30 cycles of 60 s at 94**°**C, 60 s at 48**°**C and 60 s at 72**°**C, and a final extension for 10 min at 72**°**C)* |
| *tet*(M)-*int* | 709,1812 | 30 s at 98**°**C followed by 30 cycles of 10 s at 98**°**C, 30 s at 61**°**C and 144 s at 72**°**C, and a final extension for 10 min at 72**°**C |
| P1 | 1853,1787 | 30 s at 98**°**C followed by 30 cycles of 10 s at 98**°**C, 30 s at 64**°**C and 145 s at 72**°**C, and a final extension for 10 min at 72**°**C |
| Ex2 | 1845, 1917 | 30 s at 98**°**C followed by 30 cycles of 10 s at 98**°**C, 30 s at 63**°**C and 81 s at 72**°**C, and a final extension for 10 min at 72**°**C |
| P2 | 1854,1855 | 30 s at 98**°**C followed by 30 cycles of 10 s at 98**°**C, 30 s at 65**°**C and 172 s at 72**°**C, and a final extension for 10 min at 72**°**C |
| Ex3 | 1854,1857 | 30 s at 98**°**C followed by 30 cycles of 10 s at 98**°**C, 30 s at 64**°**C and 50 s at 72**°**C, and a final extension for 10 min at 72**°**C |
| P3 | 1856,1857 | 30 s at 98**°**C followed by 30 cycles of 10 s at 98**°**C, 30 s at 63**°**C and 179 s at 72**°**C, and a final extension for 10 min at 72**°**C |
| P4 | 1858,1859 | 30 s at 98**°**C followed by 30 cycles of 10 s at 98**°**C, 30 s at 63**°**C and 45 s at 72**°**C, and a final extension for 10 min at 72**°**C |
| P5 | 1860,1859 | 30 s at 98**°**C followed by 30 cycles of 10 s at 98**°**C, 30 s at 66**°**C and 177 s at 72**°**C, and a final extension for 10 min at 72**°**C |
| P6 | 1861,1845 | 30 s at 98**°**C followed by 30 cycles of 10 s at 98**°**C, 30 s at 66**°**C and 167 s at 72**°**C, and a final extension for 10 min at 72**°**C |
| UP*tet*(M)-*int* | 526-1837 | 30 s at 98**°**C followed by 30 cycles of 10 s at 98**°**C, 30 s at 60**°**C and 145 s at 72**°**C, and a final extension for 10 min at 72**°**C |
| Ex4 | 1916,1845 | (2 min at 94**°**C followed by 30 cycles of 30 s at 94**°**C, 30 s at 63**°**C and 40 s at 72**°**C, and a final extension for 5 min at 72**°**C)* |
| P7 | 1917,1845 | 30 s at 98**°**C followed by 30 cycles of 10 s at 98**°**C, 30 s at 63**°**C and 81 s at 72**°**C, and a final extension for 10 min at 72**°**C |
| Tn916xis screening | 327,328 | (3 min at 94**°**C followed by 30 cycles of 60 s at 94**°**C, 60 s at 45**°**C and 60 s at 72**°**C, and a final extension for 10 min at 72**°**C)* |
| *tet*(M) screening | 266,267 | (3 min at 94**°**C followed by 30 cycles of 30 s at 94**°**C, 30 s at 48**°**C and 60 s at 72**°**C, and a final extension for 5 min at 72**°**C)* |

* DNA Taq polymerase (Ampliqon, Denmark) was used

Supplementary Table S3: Primers used in this study

| Primer | Sequence | Reference |
| --- | --- | --- |
| intcw459-2 (1812) | 5'-GTCCATACGTTCCTAAAGTCGTC-3' | (de Vries et al., 2009) |
| TetM sekvens 6 (709) | 5'-TCGAGGTCCGTCTGAAC-3' | (de Vries et al., 2009) |
| F1F (1835) | 5'-CGTGCAAATCTAGGTTATG-3' | This study |
| F2F (1836) | 5'-CATGAAGGAGTGTAAAGAATGA-3' | This study |
| F2R (1837) | 5'-GTGTCTTATACCATGGAAGGA-3' | This study |
| F3F (1838) | 5'-GAGCCTCTTTAATCGCT-3' | This study |
| F3R (1839) | 5'-CATATTCGTCTGTCATGC-3' | This study |
| F4 (1840) | 5'-GCTAGTGCTTCCATTAAGGA-3' | This study |
| F7F (1841) | 5'-CCATCGAATCAGAATCC-3' | This study |
| F7R (1842) | 5'-TCAGGTGGTCCAAATTC-3' | This study |
| alternativ Tet(M)-reverse (1787) | 5'-CATATGTCCTGGCGTGTCTA-3' | This study |
| tn5801ringR2 (1845) | 5'-CAATGTTCCCTTCACCAGC-3' | This study |
| F5801-1 (1853) | 5'-CGTTCCACTGACCTAGTGG-3' | This study |
| F5801-2 (1854) | 5'-CGGATACCTTGATAAGTAGCGTC-3' | This study |
| R5801-2 (1855) | 5'-ACCTAATCGTTGCCCGA-3' | This study |
| F5801-3 (1856) | 5'-AGATACACCCTCGAAGTGCT-3' | This study |
| R5801-3 (1857) | 5'-TGAGTAGTGCTCCGTCAATC-3' | This study |
| F5801-4 (1858) | 5'-GCTGGTGAAGGGAACATTG-3' | This study |
| R5801-4 (1859) | 5'-TGTCATGAAGCAATGGCA-3' | This study |
| F5801-5 (1860) | 5'-GAAGGCGAGCTCGAACA-3' | This study |
| F5801-6 (1861) | 5'-GCAATGGTGAGTTCAAGCA-3' | This study |
| fCDS3-5801(1916) | 5'-CTCGAGATGGTATCACACTTACTACAG-3' | This study |
| fCDS2-5801 (1917) | 5'-CAGGCAATCCCATCAGAAC-3' | This study |
| tet M upstream (526) | 5'-TTGAATGGAGGAAAATCAC-3' | (Agerso et al., 2006) |

Supplementary Table S4: Lists of DNA sequences obtained by PCR and the Sanger sequencing Method and used with the *Guidance only reads* option in the de Novo assembly for *E. faecium* CICYT-205

| PCR product | PCR primers: (sequencing primers)* | GenBank accession number |
| --- | --- | --- |
| UP*tet*(M)-*int* | 526-1837: (540, 526, 324, 525, 266, 307, 1756 *) | KU097342 |
| Ex2 | 1845-1917 (1917) | KU097343 |
| Ex3 | 1854-1857: (1854, 1857) | KU097344 |
| Ex4 | 1845-1916:(1845, 1916) | KU097345 |
| *int*_Tn_*_5801_* (Int459) | 1811-1812: (1811, 1812) | KU097346 |
| P4 | 1858-1859: (1858) | KU097347 |

* See Supplementary Table S2 and S3

Supplementary Table S5: Results for the assembly

| Strains |  | Selected contig measurements (including scaffolded regions) | | | |
| --- | --- | --- | --- | --- | --- |
|  | N50 contig* (bp) | Min/max contig length (bp) | Average contig length (bp) | No. of contigs/ no of contigs with average coverage >30x | Total sequence count (bp) |
| *S.* *pseudintermedius* (9841787-1) | 203.432 | 507/ 384.909 | 54.573 | 47/39 | 2.564.910 |
| *S.* *pseudintermedius* (9841998-1) | 142.279 | 548/ 274.040 | 49.388 | 51/50 | 2.518.792 |
| *S.* *pseudintermedius*  (200108299-1) | 244.448 | 593/ 640.248 | 91.796 | 27/26 | 2.478.497 |
| *S.* *aureus*  (1680) | 158.454 | 538/ 503.060 | 70.277 | 42/38 | 2.951.648 |
| *E. faecium*  (CICYT-205) | 79.891 | 1.013/ 258.231 | 34.590 | 76/75 | 2.628.853 |

* The N50 contig set is calculated by summarizing the lengths of the biggest contigs until you reach 50 % of the total contig length.

Supplementary Table S6: Antibiotic resistance genotype profiles in isolates sequenced in the study^a^

|  | Aminoglycoside | Beta-lactam | Phenicol | Fluoro-quinolone | MLS (Macrolide-Lincosamide-StreptograminB) | Tetracycline |
| --- | --- | --- | --- | --- | --- | --- |
| *S. pseudintermedius*  *98-41998-1* | *aph(3')-III; ant(6)-Ia* | *blaZ* |  |  | *erm(B)* | *tet(M)* |
| *S. pseudintermedius 98-41787-1* | *aph(3')-III; ant(6)-Ia* | *blaZ* | *cat(pC221)*  (98.77%) |  | *erm(B)* | *tet(M)* |
| *S. pseudintermedius*  *2001-08299-1* |  | *blaZ* (95.74%) |  |  |  | *tet(M)* (99.95%) |
| *E. faecium*  *CICYT-205* | *aadE;*  *aac(6')-Ii* (99.82%) |  |  |  | *msr(C)* (98.92%);  *erm(B)* (99.86%);  *lnu(B)* (99.88%); | *tet(M);*  *tet(L)* |
| *S.aureus*  *1680* | *Spc* | *mecA;*  *blaZ* |  | *norA*  (91.51%) | *erm(A)* | *tet(K);*  *tet(S);*  *tet(38)*(99.85%) |

^a^Antibiotic resistance profile detected in the WGS-sequences (assembled contigs) using the ResFinder tool^[[1]](#footnote-1)^ (%ID threshold: 80%, minimum length: 80%)

Supplementary Table S7: *tet*(M) sequence allele types and *tet*(M)-gene sequence detected in *S. pseudintermedius* in this study

| *tet*(M) sequence allele type | *S. pseudintermedius* strain(s) | GenBank accession number |
| --- | --- | --- |
| *tet*(M)_Tn_*_5801_* | 9841998-1; 9907249-2; 9841787-1; 200506768-1; 200307869-1 | KU097348 |
| *tet*(M)_Tn_*_5801-_*_like type 1_ | 200108299-1; 200506729-1 | KU097349 |
| *tet*(M) _Tn_*_916_* | 200307768-1; 200108050-1; 200506416-1; 9906237-1 | KU097350 |
| *tet*(M) _Tn_*_916-like_* _type 1_  (contained a 3 bp deletion compared to Tn*916*-like *tet*(M)) | 20007910-1 | KU097351 |
| *tet*(M) _Tn_*_916-like_* _type 2_ | 200108127-3 | KU097352 |

Supplementary Table S8: Tn*5801*-*like* elements detected in GenBank. (For species/strains marked with bold, elements were used in comparative sequence analysis together with elements sequenced in this study).

| Species/strain | Year | Country | Host/source | Reference/  accession no. |
| --- | --- | --- | --- | --- |
| *Clostridium perfringens* CW459 (partial sequenced) | Before 1977 | Farm in Wisconsin; US | Porcine faeces | PMID: 11320127  /AF329848 |
| ***Enterococcus faecalis* 62** | 2002 | (Norway) | Stool sample from healthy Norwegian infant | PMID: 21398545; 19393078  /CP002491.1; NC_017312.1 |
| *Enterococcus faecium*/ E1636 (contig00073 partial) | 1961 | The Nederland’s | Human/ bloodstream infection | PMID: 20398277  /NZ_ABRY01000071; NZ_ABRY00000000 |
| ***Enterococcus faecium* Aus0004** | 1998 | Australia, Melbourne | Human patient, blood | PMID: 22366422  /NC_017022 |
| *Enterococcus faecium* E4453 (partial contigs) | 2008 | Nederland’s | Dog/faeces | PMID: 22363425/ AEDZ01000088; AEDZ01000000 |
| *Enterococcus faecium* TX0133C | 2006 | USA, Texas | Clinical isolate, bloodstream infection | PMID: 22363425; 17968832  NZ_AEBG01000099; NZ_AEBG00000000 |
| *Enterococcus faecium* TX0133A | 2007 | USA, Texas | Clinical isolate, bloodstream infection | PMID: 22363425; 17968832  /NZ_AECH01000014; NZ_AECH00000000 |
| ***Enterococcus faecium* TX0133B** | 2006 | USA, Texas | Clinical isolate, bloodstream infection | PMID: 22363425; 17968832  /NZ_AECI01000003; NZ_AECI00000000 |
| *Lactobacillus sakei* Rits (partial sequenced) |  | Italy | Cheese | PMID: 18192429  /EF605269 |
| ***Lactococcus garvieae* IPLA 31405** | 2008 | Spain: Asturias | Spanish traditional cheese (Casin) | PMID: 22933752/ AKFO01000017; AKFO01000000 |
| ***Staphylococcus aureus* Mu50 (CC5)** | 1997 | Japan | Pus of a Japanese male baby | PMID: 11418146, 15141945  /NC_002758 |
| *Staphylococcus aureus* Mu3(CC5) | 1996 | Japan | Purulent sputum of a Japanese patient with pneumonia after surgery who had failed vancomycin therapy | PMID: 17954695; 9400512; 15141945  /NC_009782 |
| ***Staphylococcus aureus* T0131** | 2006 | China, Tianjin | Human/ 87 year-old patient | PMID: 21551295  /NC_017347 |
| ***Staphylococcus aureus* TW20** | 2003 | UK, London | Human, bacteremia | PMID: 19948800  /NC_017331 |
| *Staphylococcus aureus* JKB6008 | 2003 | New Zealand | Patient bloodstream (42 days of vancomycin treatment) | PMID: 20802046  /NC_017341 |
| *Staphylococcus aureus* Bmb9393 | 1993 | Brazil, Rio de Janeiro | Patient with blood stream infection | PMID: 23929475  /CP005288 |
| *Staphylococcus aureus* strain 2395 USA500 | 1996 | US, New York City | Clinical isolate/ wound infection | PMID: 24962815; 9652436  /CP007499 |
| ***Staphylococcus pseudintermedius* ED99** |  | Scotland, UK | Dog with bacterial pyoderma | PMID: 21398539; 22919635  /NC_017568 |
| ***Streptococcus agalactiae* COH1** | Before 1985 | US | Human | PMID: 16172379  /NZ_AAJR01000021 NZ_AAJR00000000 |
| ***Streptococcus mitis* B6** | 1994 | Germany | Human | PMID: 20195536/ NC_013853 |

### References

Agerso, Y., Pedersen, A. G., and Aarestrup, F. M. (2006). Identification of Tn*5397*-like and Tn916-like transposons and diversity of the tetracycline resistance gene *tet*(M) in enterococci from humans, pigs and poultry. *J.Antimicrob.Chemother.* 57, 832–839.

Jurado-Rabadán, S., de la Fuente, R., Ruiz-Santa-Quiteria, J. a, Orden, J. a, de Vries, L. E., and Agersø, Y. (2014). Detection and linkage to mobile genetic elements of tetracycline resistance gene *tet*(M) in *Escherichia coli* isolates from pigs. *BMC Vet. Res.* 10, 155. doi:10.1186/1746-6148-10-155.

Macrogen (2008). *http://www.macrogen.com/eng/sequencing/sequence_main.jsp*. Available at: http://www.macrogen.com/eng/sequencing/sequence_main.jsp.

De Vries, L. E., Christensen, H., Skov, R. L., Aarestrup, F. M., and Agerso, Y. (2009). Diversity of the tetracycline resistance gene *tet*(M) and identification of Tn*916*- and Tn*5801*-like (Tn*6014*) transposons in *Staphylococcus aureus* from humans and animals. *J.Antimicrob.Chemother.* 64, 490–500.

1. Center for Genomic Epidemiology Antibiotic resistance finder tool - ResFinder 2.1: <https://cge.cbs.dtu.dk/services/ResFinder–2.1/> [↑](#footnote-ref-1)
